# Identification of a phenyl ester covalent inhibitor of caseinolytic protease and analysis of the ClpP1P2 inhibition in mycobacteria

**DOI:** 10.1002/mlf2.12169

**Published:** 2025-04-15

**Authors:** Genhui Xiao, Yumeng Cui, Liangliang Zhou, Chuya Niu, Bing Wang, Jinglan Wang, Shaoyang Zhou, Miaomiao Pan, Chi Kin Chan, Yan Xia, Lan Xu, Yu Lu, Shawn Chen

**Affiliations:** ^1^ Global Health Drug Discovery Institute Beijing China; ^2^ Beijing Key Laboratory of Drug Resistance Tuberculosis Research, Beijing Tuberculosis and Thoracic Tumor Research Institute, and Beijing Chest Hospital Capital Medical University Beijing China

**Keywords:** caseinolytic protease, chemical–genetic interaction, drug combination, enzyme inhibitor, mycobacteria

## Abstract

The caseinolytic protease complex ClpP1P2 is crucial for protein homeostasis in mycobacteria and stress response and virulence of the pathogens. Its role as a potential drug target for combating tuberculosis (TB) has just begun to be substantiated in drug discovery research. We conducted a biochemical screening targeting the ClpP1P2 using a library of compounds phenotypically active against *Mycobacterium tuberculosis* (Mtb). The screening identified a phenyl ester compound GDI‐5755, inhibiting the growth of Mtb and *M. bovis* BCG, the model organism of mycobacteria. GDI‐5755 covalently modified the active‐site serine residue of ClpP1, rendering the peptidase inactive, which was delineated through protein mass spectrometry and kinetic analyses. GDI‐5755 exerted antibacterial activity by inhibiting ClpP1P2 in the bacteria, which could be demonstrated through a minimum inhibitory concentration (MIC) shift assay with a *clpP1* CRISPRi knockdown (*clpP1*‐KD) mutant GH189. The knockdown also remarkably heightened the mutant's sensitivity to ethionamide and meropenem, but not to many other TB drugs. On the other hand, a comparative proteomic analysis of wild‐type cells exposed to GDI‐5755 revealed the dysregulated proteome, specifically showing changes in the expression levels of multiple TB drug targets, including EthA, Ldt_Mt2_, and PanD. Subsequent evaluation confirmed the synergistic activity of GDI‐5755 when combined with the TB drugs to inhibit mycobacterial growth. Our findings indicate that small‐molecule inhibitors targeting ClpP1P2, when used alongside existing TB medications, could represent novel therapeutic strategies.

## Impact statement

To combat the rising challenge of drug‐resistant *Mycobacterium tuberculosis* (Mtb), it is imperative to identify novel small‐molecule compounds through unexplored antibacterial mechanisms. The ClpP1P2 protease offers a promising avenue for tuberculosis (TB) drug development. In this study, we identified an inhibitor of the ClpP1P2 enzyme, GDI‐5755, through screening with a specialized compound library. GDI‐5755 showed a synergistic interaction with a few TB therapeutics, enhancing their ability to suppress mycobacterial growth. The findings underscore the value of exploring the cellular mechanisms of bioactive molecules, offering a compelling strategy for formulating innovative TB drug regimens.

## INTRODUCTION

Tuberculosis (TB) is a potentially serious infectious disease caused by a single etiologic agent, *Mycobacterium tuberculosis* (Mtb). Despite being a preventable and curable disease, TB remains one of the top disease killers worldwide, resulting in ~10 million active cases and ~1.3 million deaths in 2022, which is an increase compared to the declining trend in the previous years^1^. The emergence of multidrug‐resistant TB (MDR‐TB) that is resistant to at least isoniazid and rifampicin, the two potent TB drugs widely prescribed, and highly drug‐resistant TB (XDR‐TB) resistant not only to the two drugs but also extensively to a group of second‐line drugs, poses significant challenges to TB treatment and control.

A typical TB treatment involves a long course of antibiotic therapy over 6 months. For MDR‐TB and XDR‐TB, the treatment becomes more prolonged, lasting up to 24 months and involving more toxic and expensive drugs that could have severe side effects. Furthermore, current TB drugs are less effective against persistent bacteria in dormant states, evading the host's immune response. These challenges have spurred the need for novel strategies in TB drug discovery, focusing on targets and compounds with new mechanisms of action to overcome drug resistance and reduce treatment duration^2^. There is also an emphasis on developing drug combination therapies to improve efficacy, prevent the development of resistance, and minimize adverse effects by leveraging potential synergistic effects between drugs. The ultimate goal of global health is to eradicate Mtb infection.

Protein degradation plays a critical role in standard bacterial physiology and the pathogenicity of pathogens. Mtb encodes a cytosolic proteasome and several proteases, one of which is the caseinolytic protease (Clp) enzyme complex. A functional Clp comprises a serine protease core interacting with an ATP‐dependent unfoldase called AAA+ chaperone^3^. The Clp core in mycobacteria has two stacked heptameric rings made up of ClpP1 and ClpP2 homo‐oligomers. ClpP1P2 is shown, through biochemical experiments, to possess standalone peptidase activity; however, its proteolytic activity toward folded proteins is very weak unless the AAA+ ATPase is present. In Mtb, the ATPase activity resides in a hexameric complex composed of either ClpC1 or ClpX subunits. This third ring of oligomers docks on the surface of ClpP2, harnessing the energy released from ATP hydrolysis to unwind and translocate a protein substrate into the catalytic chamber extending from ClpP2 to ClpP1. The mycobacterial Clp system, as a whole, is structurally and functionally asymmetric; the proteolytic activity of ClpP1P2 is regulated by the protein dynamics, pH in the chamber, and ClpX or ClpC1 (so‐called regulatory ATPases). The protein degradation pathway through the ClpP1P2 peptidase is thought to be required in quality control and turnover of proteins that impact mycobacterial growth, survival, or re‐emergence from dormancy^4^, ^5^, ^6^, ^7^, ^8^, ^9^, ^10^. Indeed, in contrast to other common pathogens, the *clpP1P2* organized as one genetic operon in Mtb is essential and vulnerable, presenting an attractive target for discovering novel antituberculosis therapeutics.

ClpP as an antimycobacterial target was genetically validated using various models such as Mtb, *M. bovis* BCG (BCG), and *Mycolibacterium smegmatis* (Msmeg). The first compound discovered acting on ClpP is the acyldepsipeptide (ADEP), naturally produced by actinobacteria *Streptomyces* ^6^, ^11^, ^12^. Synthetic ADEP derivatives were later found to kill mycobacteria by preventing the interaction of ClpP1P2 with ClpC1 or ClpX^7^. In vitro reconstitution of the proteolytic activity of Mtb ClpP1P2 facilitated the characterization of its peptidase reaction and specific cleavage sites^13^. Peptidyl boronates, which mimic substrates, were discovered as potent catalytic site inhibitors of Mtb ClpP1P2, with some displaying anti‐Mtb activity^14^. The peptidyl boronate drug bortezomib was identified as a Clp protease inhibitor through a whole‐cell screening using an engineered Msmeg reporter strain^15^; however, it is unclear whether the antimycobacterial activity results mostly from ClpP inhibition^16^. Biochemical screening of the ClpP1P2 against a commercial compound library identified another drug Cediranib, which inhibits Mtb ClpP1P2 enzyme activity by binding to an allosteric pocket of ClpP1 subunit^17^. Recently, target‐based genome mining of actinobacteria has uncovered new natural products of bicyclocarbamate and β‐lactone families, capable of covalently modifying the active‐site serine residue of the distinctive ClpP enzymes in various microorganisms^18^, ^19^. While the antimicrobial spectrum of these compounds remains to be determined, they are anticipated to inhibit Mtb ClpP1P2. Previous studies have conducted omics‐based analysis in Mtb by knocking down *clpP1P2* expression^7^, and yet, the proteomic response to small‐molecule ClpP1P2 inhibitors in slow‐growing mycobacteria is unexplored. Research in this area could guide the development of effective drug combinations that include a ClpP1P2 inhibitor, potentially enhancing treatments against mycobacterial pathogens such as Mtb.

In this study, we conducted a biochemical screening of a library of TB whole‐cell‐active compounds to identify inhibitors of the ClpP1P2 complex. A phenyl ester compound, GDI‐5755 (CID: 4812655 in the PubChem), was found to covalently modify the ClpP1 protein. We monitored the growth inhibition with BCG in the presence of GDI‐5755 and conducted a proteomic analysis to identify the up‐ and downregulated proteins. A *clpP1* CRISPRi knockdown (*clpP1*‐KD) mutant was used to demonstrate that GDI‐5755 targets ClpP in mycobacteria, and this strain was used to predictively screen for synergistic effects between ClpP inhibition and 15 clinically relevant antibiotics. The results were utilized to formulate drug combinations that could significantly decrease the MIC of certain TB drugs.

## RESULTS

### High‐throughput screening for the Mtb ClpP1P2 inhibitor and the biochemical characterizations of GDI‐5755

**Figure 1:**
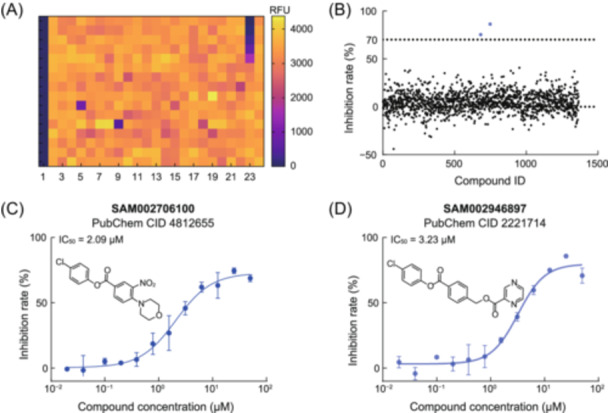
The 1364 compounds from the whole‐cell‐active library screened with the Mtb ClpP1P2 peptidase assay.  (A) A heatmap displaying the relative fluorescence of one assay plate. Bortezomib (50 µM in column 1 and a serial dilution from 50 µM in column 23) and DMSO (0.5% in columns 2 and 24) were the controls. The compounds were in columns 3 to 22. Fluorescence signals are illustrated in light yellow (baseline) and dark purple (inhibition).  (B) Two inhibitory hits identified by the screening, reducing over 70% of the peptidase activity (dotted line) at 50 μM. (C, D) IC_50_ values of the inhibitors SAM002706100 (C) and SAM002946897 (D) determined with library stocks, at 2.09 and 3.23 μM, respectively.

Akopian and co‐workers are the first to report the enzymatic study of Mtb Clp^13^, where the peptidase assay was established. Purified ClpP1 and ClpP2 proteins can be reconstituted into the active form of hetero‐tetradecamer in the presence of an N‐blocked dipeptide aldehyde substrate mimic. In vitro inhibition of ClpP1P2 peptidase activity is measured by monitoring the cleavage of a fluorogenic substrate tripeptide Ac‐PKM‐amc to release quenching of the fluorescence signal. We were able to optimize the assay into a 384‐well format and screen against a library of Mtb whole‐cell‐active compounds (Figure 1A). The 1364 compounds, kindly provided by the Global Alliance for TB Drug Development, were selected based on the data from historical phenotypical screening campaigns^20^, ^21^. The prior information is now available in PubChem^22^, but the mechanism and molecular targets of most compounds are unknown. ClpP1P2 biochemical screening of the library compounds at 50 µM produced two hits inhibiting the peptidase by more than 70% (Figure 1B). Cherry‐picking the top hits from the library stocks confirmed the inhibitory activity; in a follow‐up concentration–response experiment, the respective half‐maximal inhibitory concentration (IC_50_) was measured at 2.09 and 3.23 µM (Figure 1C,D).

**Figure 2:**
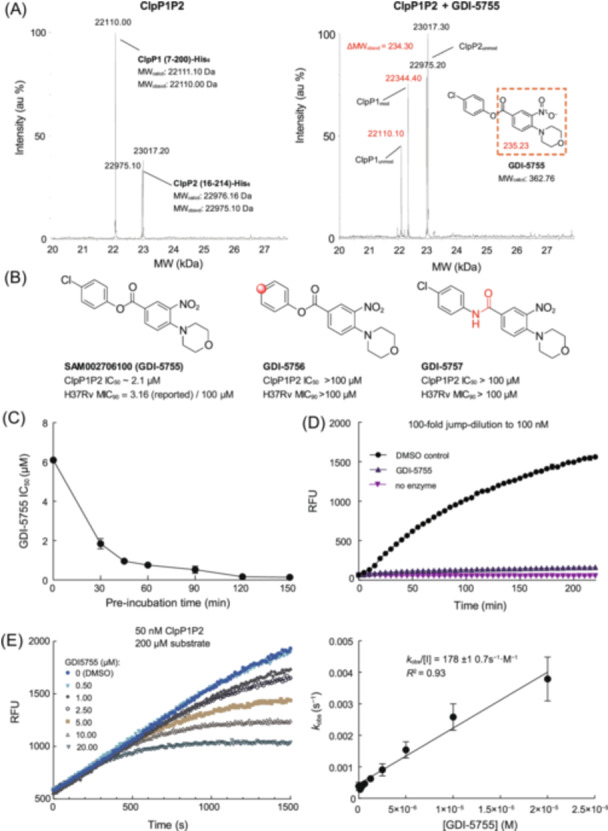
Biochemical characterization of the ClpP1P2 inhibition with a resynthesized inhibitor and the measured activities of GDI‐5755 and its analogs. (A) Intact protein mass spectrometry reveals that GDI‐5755 covalently modifies the activated ClpP1P2 protein complex. The overexpressed and purified ClpP2 with MW at 23017.30 Da may have an N‐terminal acetyl group. The subscript after MW indicates either the calculated (calcd) or observed (obsd) MW. The subscript after the name of a ClpP subunit indicates a modified (mod) or unmodified (unmod) protein. (B) Structures and activities of neat GDI‐5755, and the synthesized analogs GDI‐5756 and GDI‐5757. (C) Plot of GDI‐5755 IC_50_ versus pre‐incubation time. The IC_50_ decreased to nearly the enzyme concentration as the time was prolonged, implying an irreversible inactivation of ClpP1P2. (D) A jump dilution analysis confirms that the fully inactivated ClpP1P2 cannot recover its peptidase activity after 100 dilution of a pre‐incubated enzyme–inhibitor mixture (See Materials and Methods section and Figure S3). (E) Progress curves of ClpP1P2 peptidase reactions with varying amounts of GDI‐5755 and the *k*_obs_/[I] determination. *k*_obs_ was calculated by fitting to Equation (2). Biochemical assays were performed at least twice with triplicates. The data points are presented as mean ± SD. RFU, relative fluorescence units.

Both the hits contain a phenyl ester core. Very few phenyl ester compounds were previously shown to be the mechanism‐based inhibitors of staphylococcal ClpP (SaClpP) through covalent modification of the active‐site serine residue^23^, ^24^. Due to the restricted access to the active site in different bacterial ClpPs, only the electrophilic phenyl ester compounds mimicking a short peptide substrate can inhibit a ClpP by either covalent or noncovalent binding. If a covalent mechanism is involved, the stability of the leaving group, which is a 4‐chlorophenol (4CP) in the two hits, would be crucial for the reactivity of the compound. The proposed reaction of a 4‐chlorophenyl ester in modifying a serine residue is shown (Figure S1). As the MICs of the hits were reported at single‐digit µM in PubChem, the more potent SAM002706100, registered as GDI‐5755 in our internal system, was synthesized for confirmation of the biochemical and cellular activities (Figure 2).

The resynthesized GDI‐5575 was tested under the assay conditions as in the screening and the IC_50_ confirmed with three replicates was 2.4 ± 0.07 μM. Intact protein mass spectrometry (MS) was used to determine the exact mass of the ClpP1P2, with or without incubation with GDI‐5755. The proteins had an observed mass of 22110.00 Da (ClpP1) and 22975.10 Da (ClpP2), respectively, in agreement with the calculated molecular weights. A new peak with 22344.40 Da mass appeared in the spectrum of the GDI‐5755‐treated sample (Figure 2A). The mass difference between this new species and ClpP1 is 234.30 Da, equal to the mass of the acyl part of GDI‐5755. The mass of the new peak agrees with the Ser98‐modified ClpP1 (Figure S1). Notably, the ClpP2 (and the pre‐existing N‐acetylated ClpP2 with 23017.20 Da mass) was unchanged within an error range after the GDI‐5755 treatment. Thus, the loss of peptide degradation activities in the inhibited reaction is most likely due to an acylation of ClpP1 by GDI‐5755. However, the freshly resynthesized GDI‐5575 only showed an MIC against H37Rv at 100 µM. The confirmed antimycobacterial MICs of GDI‐5755 were much higher than the reported 3.16 µM in the PubChem database. Two analogues, GDI‐5756 and GDI‐5757 (Figure 2B), were synthesized to probe the activities. GDI‐5756 has a phenyl group without electron‐withdrawing substitution, making the ester bond less reactive than GDI‐5755. GDI‐5757 has the ester replaced with a stable amide bond. Neither analog could inhibit ClpP1P2 peptidase or Mtb whole cell at 100 µM. It is reasoned that, after binding to ClpP1, the intrinsic reactivity of GDI‐5755 causes the inactivation of ClpP1.

The peptidase assay was modified to investigate the GDI‐5755 inhibition. First, ClpP1P2 was pre‐incubated with the resynthesized neat GDI‐5755 at various concentrations before adding a substrate. As the pre‐incubation time increased, the measured IC_50_ of GDI‐5755 decreased to nearly enzyme concentration (e.g., ~0.13 µM) (Figure 2C). However, the enzyme pre‐incubated with solvent DMSO lost some activity during the prolonged incubation (Figure S2). The reversibility of ClpP inactivation was then assessed using a jump dilution method. ClpP1P2 was incubated with GDI‐5755 (or the solvent as a control) at 10 µM for 1 h. The inhibitor concentration was more than 20‐fold higher than the IC_50_; the enzyme was expected to be completely inhibited within this time. Afterward, the mixture was diluted 100‐, 200‐, or 400‐fold with buffer containing the substrate, and the recovery of enzyme activity was measured. It was observed that the ClpP1P2 pre‐incubated with DMSO and diluted down to 25 nM maintained a reasonable level of activity, but the ClpP1P2 inactivated by GDI‐5755 and diluted to 100 nM did not show recovery of peptidase activity in a 4‐h course (Figures 2D and S3). Interestingly, as the MS showed that GDI‐5755 does not modify ClpP2, the loss of peptidase activity could be attributed to disorganizing ClpP1 oligomer and disassembly of the complex, or the ClpP2 does not contribute to the peptidase activity in vitro like that observed with genetic experiments^9^. The biochemical data suggest that GDI‐5755 is a fast‐on inhibitor, irreversibly inactivating ClpP1P2. Finally, the inactivation efficiency *k*_obs_/[I] of GDI‐5755 was determined to be 178 ± 10.7 s^−1^ M^−1^ (Figure 2E), indicating that it is inferior to the phenyl ester inhibitors of SaClpP but better than the synthetic β‐lactone inhibitors^23^. Although there is room for improving GDI‐5755's inhibitory activity, the limited stability of the phenyl ester compound^25^ generally precludes it from being developed into an oral drug. Nonetheless, it could be a cellular probe for a study of ClpP‐dependent pathways in mycobacteria.

### Use of *clpP*‐KD mutants for analysis of the target engagement of GDI‐5755

**Figure 3:**
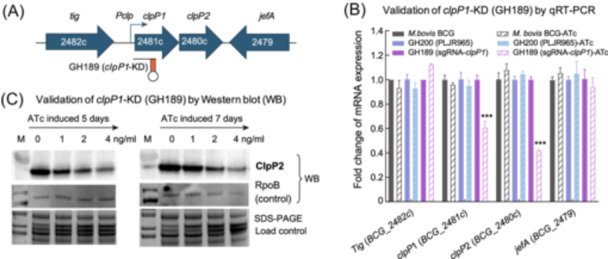
Construction of the *clpP1*‐KD strain GH189 and confirmation with Western blot and qRT‐PCR. (A) Location of sgRNA targeting sequences in the *clpP* operon in BCG. *clpP1* and *clpP2* are co‐transcribed under a single promoter. Orientations of the adjacent genes *tig* (*BCG_2482c*) and *jefA* (*BCG_2479*) are shown. (B) qRT‐PCR assays performed to confirm the relative mRNA expression levels of *clpP1P2* and adjacent genes, normalized to *rpoB* at 5 days post‐induction with or without 20 ng/ml ATc. GH200 is a strain carrying the control plasmid. All data points represent the means ± SD of two biological replicates and are representative of at least two independent experiments. (C) Western blot showing the ClpP2 protein level decreased in GH189 in an ATc dose‐dependent manner at 5 days and 7 days after the ATc induction, compared to the control bands detected with the RpoB antibody.

The inducible CRISPR interference (CRISPRi) approach has been performed in Mtb and the fast‐growing Msmeg to create *clpP1*‐KD and *clpP2*‐KD mutants^9^, ^26^. The KD mutants were used to demonstrate the essentiality of *clpP* genes and investigate the functions of ClpP1P2 in mycobacteria. In a target‐based antibacterial discovery, a gene knockdown mutant is also commonly used to analyze the specificity and selectivity of compounds in the cellular context. We created *clpP*‐KD strains using the vaccine strain BCG. BCG is a surrogate model of slow‐growing mycobacteria and can be used in a biosafety level‐2 lab to facilitate early drug discovery research. *M. bovis* is also a globally prevalent animal pathogen; its genome shares 99.95% sequence homology with other members of Mtb complex (MTBC) lineages^27^, ^28^. The *clpP* locus in BCG including the promoter sequences is identical to Mtb. The *clpP1P2* are in one transcriptional unit and the ORFs have overlapping stop and start codons (Figure 3A).

**Figure 4:**
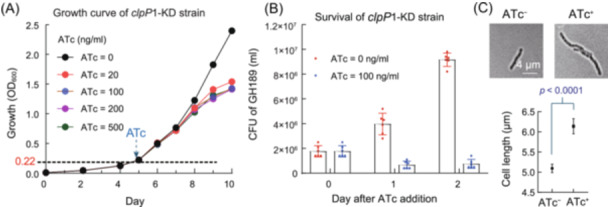
Impact of *clpP1‐*KD on bacterial growth, survival, and cell morphology. (A) Growth kinetics of GH189 reflected by the optical density (OD_600_) of the culture 10 days post‐induction with various concentrations of ATc. (B) Assessment of the viability of GH189 by counting the colony‐forming units (CFUs) on 7H10 agar plates on day 1 or day 2 after ATc induction. The culture reached the logarithmic phase (OD_600_ = 0.22), marked as point (A), at the time of induction. (C) The cell morphology recorded on day 1 post‐induction with or without ATc. The error bar shows the standard error of the mean. Unpaired two‐tailed *t*‐tests obtained *p* < 0.0001.

Construction of the CRISPRi BCG strains, expressing the *clpP1‐ or clpP*2‐targeting sgRNA and scrambled sgRNA (sgRNA‐control), was performed the same way as with Mtb^26^. For simplicity, we focused on the *clpP*1‐KD strain GH189 and analyzed ClpP2, the product of the second gene in the operon. Confirmation of the reduced *clpP* transcription in GH189 before and after anhydrotetracycline (ATc) induction using proper controls is shown in Figure 3B. Western blot analysis experiments demonstrated a decrease in the ClpP2 protein level in an ATc dose‐dependent manner 5–7 days after ATc induction at the time of inoculation (Figure 3C). To investigate the effects of suppressing *clpP* post‐transcriptionally, the interference was induced with varying concentrations of ATc just before the exponential growth phase (Figure 4A). Bacterial growth curves revealed that the knockdown brought about a change 2 days after the induction. Additionally, the colony‐forming unit (CFU) of the GH189 culture decreased twofold 1 day after the induction (Figure 4B). Cell morphology analysis with GH189 at this point demonstrated that the cell length was significantly longer (Figure 4C), in agreement with previous observations in the *clpP*‐KD of other mycobacteria^9^, ^29^. The essentiality of *clpP1P2* in BCG is thus genetically validated; the encoded enzyme is a vulnerable drug target in this mycobacterial pathogen.

**Figure 5:**
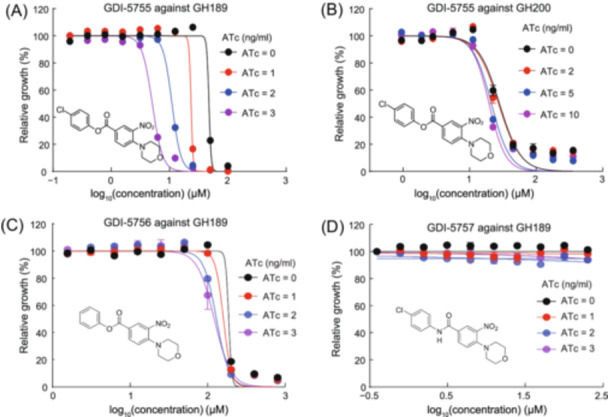
Validation of GDI‐5755 target engagement using the *clpP1*‐KD strain GH189. (A, B) GDI‐5755 against GH189 (A) and GH200 (B). GH189 showed increasing susceptibility to GDI‐5755 after *clpP1*‐KD was induced. The extent of GDI‐5755s MIC shifting to smaller values was co‐related to the increasing concentration of ATc in the medium, which should control the expression levels of *clpP1*‐targeting sgRNA in GH189. In contrast, when tested against a control strain GH200 (B), in which scrambled sgRNA (sgRNA control) expression was induced, the MIC of GDI‐5755 did not change. (C, D) The closely related analogs, GDI‐5756 (C) and GDI‐5757 (D), shown to be much less active in the enzymatic assay, causing marginal or no MIC shifting when tested against GH189, respectively. Each experiment was performed three times independently and a representative dataset is shown.

To analyze the target engagement of the screened compounds in whole cells, the growth was measured using the resazurin reduction microplate assay. As shown in Figure 5A, GH189, expressing the *clpP1*‐targeting sgRNA and with increasingly less ClpP on ATc induction, displayed increasing susceptibility to GDI‐5755; the MIC against GH189 decreased from 52.1 to 7.9 µM. The magnitude of MIC down‐shifting was dependent on the concentration of inducer ATc. MIC of GDI‐5755 against a control strain GH200, expressing the scrambled sgRNA upon the induction, did not show shifting (Figure 5B). In contrast, when the other two GDI‐5755 derivatives that appeared inactive in biochemical assays were tested against GH189, the MIC of GDI‐5756 marginally shifted down and GDI‐5757 at the highest soluble concentration remained inactive (Figure 5C,D). This experiment demonstrated that the inhibitory activity of GDI‐5755 is mostly and directly associated with ClpP1 inhibition in mycobacteria.

### Proteome and the anti‐TB target proteins in GDI‐5755 inhibited cells

**Figure 6:**
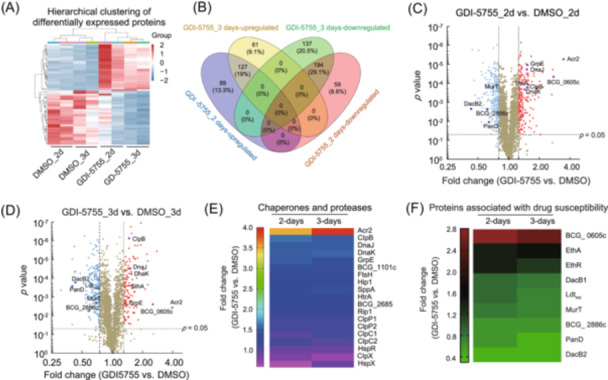
iTRAQ‐based proteomics analysis of GDI‐5755‐treated wild‐type BCG cells. (A) A heatmap showing the normalized read counts of differentially expressed proteins (DEPs) in two biological replicates of GDI‐5755 treatment at twofold MIC for 2 days (2 d) or 3 days (3 d), compared to the controls of the DMSO (solvent) treatment. (B) A comparative Venn diagram illustrating the overlapping and unique protein alterations detected by the iTRAQ approach at the time indicated. (C, D) Volcano plot presentations with protein samples extracted at Day 2 or Day 3. *p* values are plotted on the *y*‐axis. Significantly DEPs (*p* ≤ 0.05, iTRAQ ≥ 1.25 or iTRAQ ≤ 0.8) are denoted by red dots or blue dots, and all others are denoted by light brown dots. (E, F) Heatmaps showing the significantly regulated proteins in the GDI‐5755‐treated group versus the DMSO‐treated group. The concerned proteins are in the proteostasis network (E) or associated with drug susceptibility (F). Color intensity indicates relative upregulation or accumulation (>1.0) or downregulation (<1.0).

The Clp system is a promising drug target, and proteomic analysis of cell growth perturbed with a ClpP inhibitor could guide antibacterial drug development^30^. Two previous proteomic studies, using different genetic approaches in Mtb to knock down *clpC1* and *clpP2* or deplete ClpP2 protein^7^, ^31^, aimed at searching for the protein substrates of the Clp system. Major transcriptional factors and many essential proteins were preliminarily identified. As GDI‐5755 has a clear on‐target cellular activity, we attempted to use this compound to explore the proteome and look for the regulation of the proteins or pathways targeted by existing TB drugs. We analyzed the impact of GDI‐5755 on the proteomes on Day 2 and Day 3 after the treatment using iTRAQ‐based quantitative proteomics. As shown in Figure 6, more proteins were downregulated in the GDI‐5755 treatment than in the DMSO control. After the 2‐day treatment (equivalent to two doubling periods) (Figure 6B), ~37.9% of the detected proteome were downregulated (Supporting Information: additional File S1), and 29.1% of the proteome remained downregulated into the third day when nearly half (49.6%) of the proteome went down. 216 proteins (89 plus 127) on Day 2 and 188 proteins (127 plus 61) on Day 3 appeared to have accumulated (upregulated) in the treated cells (Figure 6B) (Supporting Information: additional File S2), which might be directly related to ClpP function. However, a pathway enrichment analysis failed to identify specific metabolic pathways that could be attributed to the growth inhibition. As previously reported with ClpC1‐depleted Mtb cells^31^, the small heat shock protein Acr2 (Hsp20 or Rv0251c) was the most accumulated protein in the treated BCG cells (Figure 6C,D). A few protein‐folding chaperonins or chaperones (DnaJ, K and GrpE) significantly accumulated. Some proteases (from BCG_1101c to Rip1 in Figure 6E) were also upregulated. These data suggest that protein folding and proteolytic pathways are mostly affected by ClpP1 inhibition. Unlike the genetics‐based studies, in both 2‐ and 3‐day GDI‐5755‐treated cells, ClpB (an AAA+ chaperone) was upregulated 1.56‐ and 1.47‐fold while ClpP1 and ClpP2 were upregulated 1.10‐ to 1.14‐fold at the two time points (Figure 6E). ClpC1 and C2 levels marginally increased; ClpX and the heat shock‐negative regulator HspR decreased. The regulatory circuit of *Clp* genes and the translation are presently largely underexplored. We also compared the two proteomes of GDI‐5755 treatment to the reported proteome of ClpP1 depletion^7^ and annotated the BCG genes encoding the proteins with the corresponding Mtb genes. The essentiality of the protein genes is marked, especially those in the downregulation list (Table S5). In another list (Table S6), among the top 20 upregulated proteins in the GDI‐5755‐treated BCG, 12 proteins reportedly accumulated in the ClpP1‐depleted Mtb^7^. Overall, the inhibition of the ClpP system by GDI‐5755 likely led to a compensatory up‐ and downregulation of proteins involved in protein homeostasis.

Among the highly accumulated proteins, immediately after the Acr2 are two methyltransferases (Supporting Information: additional File S1), one of which is BCG_0605c, whose sequence is 100% identical to Rv0560c of Mtb. Rv0560c was implicated in drug resistance, shown to transfer a methyl group from cofactor *S*‐adenosylmethionine to a secondary amine in a mycobactericidal compound or a hydroxyl group in a bacterial small‐molecule toxin^32^, ^33^. GDI‐5755 could not be modified, but the product 4CP could be modified by this type of methylation. The mode of action of 4CP is the uncoupling of oxidative phosphorylation. It inhibits microbial growth at a high concentration^34^. Alternatively, BCG_0605c may perform a function through interaction with EthR^35^. EthR is a ligand‐bound transcriptional repressor^36^ acting on a DNA operator at the *ethR‐ethA* locus. EthA is a Bayer–Villiger monooxygenase thought to be involved in mycolic acid metabolism. EthA can catalyze the metabolism of thiocarbamide‐containing compounds and activate the prodrug ethionamide (ETH)^37^, ^38^. EthA was consistently upregulated >1.4‐fold in GDI‐5755‐treated cells, and so was the EthR (Figure 6F). The transcriptional regulations of *BCG_0605c* ^39^ and *ethA‐ethR* ^38^ can be complicated. Here, it is only concluded that the accumulation of these proteins is a response to the GDI‐5755 treatment.

The upregulation of EthA prompted us to search the datasets for other drug targets (Figure 6F). Aspartate decarboxylase PanD, which is the target of another prodrug pyrazinamide (PZA)^40^, ^41^, ^42^, was markedly downregulated; the amount of PanD in GDI‐5755‐treated cells was only 40‐60% of that in the DMSO control. Additionally, several cell wall biosynthesis enzymes were downregulated, including penicillin‐binding proteins DacB1 and DacB2, l,d‐transpeptidase Ldt_Mt2,_ and a penicillin‐binding lipoprotein BCG_2886c (equivalent to Rv2684c)^43^, ^44^. β‐lactam drugs, particularly the meropenem (MPM) approved for TB treatment^45^, should have some activity against these enzymes. The findings led to a hypothesis that GDI‐5755 could be used as a potentiator in combination with one of the anti‐TB drugs: ETH, PZA, and MPM.

### Chemical–genetic interaction study for the effect of *clpP1*‐KD on drug MICs

While the proteomic studies were performed, we used the *clpP1*‐KD hypomorph strain GH189 to predictively screen for synergistic effects between *clpP1*‐KD and 14 antibiotics (Table 1). Downshifts >2‐fold in the MIC of ETH, MPM, or ethambutol (EMB) against GH189 were noted. This indicated that *M. bovis* cells became more susceptible to the three antibiotics during or after *clpP1*‐KD. In contrast, the transcriptional knockdown of *clpP1P2* did not affect the efficacy of the other 11 antibiotics. Because *M. bovis* is naturally resistant to PZA^46^, we could not study the interaction of *clpP1*‐KD and PZA in GH189. We also tested the five drug compounds in the biochemical assay of ClpP1P2 and found that none of them inhibited the peptidase activity at the highest concentration (Figure S4). The results suggest that the protein degradation pathway via ClpP intersects with other essential cellular pathways, which can be leveraged for designing a synergistic two‐drug combination, one of which is a ClpP inhibitor.

**Table 1:** Effect of *clpP1*‐KD on the MIC of antitubercular drugs against the GH189.

TB Drug	MIC (µg/ml) when no *clpP1*‐KD	MIC (µg/ml) when *clpP1*‐KD induced	TB Drug	MIC (µg/ml) when no *clpP1*‐KD	MIC (µg/ml) when *clpP1*‐KD induced
Pretomanid	0.12	0.06	Rifampicin	0.01	0.01
Azithromycin	0.5	0.46	Isoniazid	0.066	0.057
d‐Cycloserine	11	9.3	Streptomycin	0.24	0.23
Capreomycin	0.25	0.14	Linezolid	1.0	0.97
Moxifloxacin	0.03	0.02	Ethambutol	0.62	0.28
Bedaquiline	0.06	0.06	Meropenem	5.3	1.6
Clarithromycin	0.11	0.10	Ethionamide	46	6.4

Each MIC measurement was performed three times independently. The MIC values underlined shifted downward 4‐ to 6‐ fold, compared to those with no *clpP1*‐KD.

### Synergistic killing of mycobacteria using the anti‐TB drugs in combination with GDI‐5755

**Figure 7:**
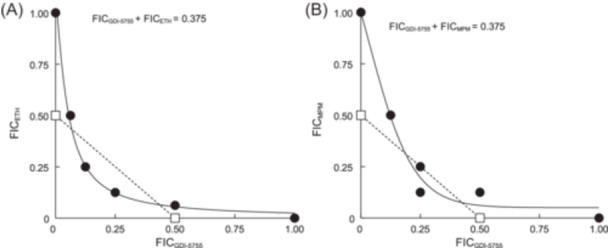
Isobolograms of GDI‐5755 in combination with ethionamide (ETH) or meropenem (MPM). (A) ETH. (B) MPM. The fractional inhibition concentration (FIC) was obtained in a checkerboard assay against BCG. The concave curvature below or near the dashed line drawn between two FICs at 0.5 indicates a synergistic interaction.

A checkboard assay was used to verify a potential synergistic effect between ClpP inhibition and an anti‐TB drug, evidenced by both proteomic and chemical–genetic interaction experiments. As illustrated in Figure 7, treatment of wild‐type BCG with ClpP‐targeting compound GDI‐5755 and ETH or MPM lowered the respective single‐drug MICs to a fraction. The fractional inhibitory concentration index (FICI) for each pair was below 0.5, which means that a combination of the ClpP inhibitor with ETH or MPM could synergistically kill slow‐growing mycobacteria in this experiment.

## DISCUSSION

TB is the world's oldest pandemic. With the rise of drug resistance, the protracted duration of treatment, and the adverse effects of existing therapies, there is a critical need for the discovery of novel therapeutic agents and innovative treatment approaches against TB. The ClpP1P2 peptidase of Mtb has gained attention as a promising target for anti‐TB drug development. Despite the interest, systematic study of small‐molecule inhibitors targeting ClpP1P2 in the slow‐growing mycobacteria is notably limited. Unraveling the mechanism of action of potential inhibitors is vital for therapeutic development. Our investigation led to the identification of two ClpP1P2 inhibitors from high‐throughput screening of a curated library containing known whole‐cell‐active compounds^22^. Following this, we chemically synthesized and validated the biochemical and cellular activity of one such inhibitor: GDI‐5755. Protein MS analysis demonstrated that GDI‐5755 covalently modifies the ClpP1 subunit. Through kinetic analysis, we found the modification irreversibly inactivated the peptidase; the inactivation efficiency of GDI‐5755 was thereby measured. To explore the cellular effects, we generated the *clpP1*‐KD mutant, GH189, in the closely related bovine pathogen BCG. This mutant facilitated the confirmation of ClpP as the molecular target of GDI‐5755 via microbiological assays, establishing that the inhibitor engages with its intended target within the cell. We further analyzed the proteomic changes in BCG upon GDI‐5755 treatment compared to ClpP‐depleted Mtb, identifying a series of proteins that are likely ClpP substrates in mycobacteria. Integrating the results from chemical–genetic interaction studies of GH189 with the proteomic data derived from GDI‐5755 treatment, we designed a putative drug combination strategy. Subsequent microbiological testing demonstrated that the ClpP1P2 inhibitor GDI‐5755 could enhance the efficacy of at least two extant TB drugs: ETH and MPM.

Entry into the active sites of the ClpP1P2 complex is modulated by its oligomerization state and interaction with the regulatory ClpC1. Library screenings have identified enzymatic inhibitors resembling substrates or peptidomimetics^15^. Covalent inhibitors present a viable avenue for the development of drugs targeting serine proteases such as ClpP1P2. GDI‐5755 exemplifies a compound with these attributes; however, because of the unshielded phenyl ester bond, GDI‐5755 is susceptible to hydrolysis in a protein‐free buffer over time (data not published). Importantly, the two structurally close analogs GDI‐5756 and GDI‐5757 (Figure 2B) lack detectable enzymatic or cellular activities, casting doubt on the potential of the GDI‐5755 scaffold for further development.

In drug discovery, it is a pivotal step to validate the antibacterial activity of screening hits through in vivo assays such as utilizing bacterial strains with inducible expression of target proteins. Such strains can show variable drug susceptibility or resistance when the target protein is knocked down or overexpressed, making them valuable for confirming the target engagement of compounds in mycobacteria. In previous studies, bortezomib was identified as a Clp protease inhibitor via target‐based whole‐cell screening using an engineered Msmeg strain with a fluorescent reporter harboring a peptide tag recognized by the Clp system^14^, ^15^. While this reporter‐based screening can facilitate the identification of inhibitors, it may not adequately discriminate between general serine protease inhibitors and those that are more specific to the Clp system. Deploying GH189 and another strain expressing ClpP2‐targeting sgRNA, which are BSL‐2‐compliant and have been thoroughly characterized by biological methods and chemical probes, will significantly advance the discovery and validation of selective ClpP1P2 inhibitors.

In this study, we applied quantitative proteomics to elucidate the cellular response to treatment with the ClpP1P2 inhibitor GDI‐5755. The resulting proteomic data indicated an accumulation or upregulation of protein chaperones involved in regulating protein folding and aggregation, as well as proteases that might act to compensate for reduced ClpP activity (Figure 6E). We highlighted three proteins with significant accumulation: BCG_0605c (identical to Rv0560c in Mtb), EthR, and EthA, all of which have been implicated in the intracellular activity or metabolism of small molecules. Table S6 lists a total of 53 proteins with increased accumulation in GDI‐5755‐treated BCG and ClpP1‐depleted Mtb, suggesting that these proteins are putative substrates of the ClpP system, including Acr2 (Hsp20), which was experimentally verified^31^. Examination of these proteins’ sequences may uncover motifs that dictate their degradation via ClpP. Conversely, Table S5 captures 43 proteins found to be downregulated in the two studies. These proteins marked as essential or associated with growth defects could have increasing vulnerability in cells with compromised ClpP function, making them attractive candidates for targeted discovery efforts. Overall, the proteomic insights gained from GDI‐5755 inhibition contribute not only to our understanding of mycobacterial biology but also to the development of novel TB treatment strategies.

The application of *clpP1*‐KD in mycobacteria formed the basis of a chemical–genetic study aimed at assessing the potential to enhance the efficacy of existing TB drugs. Our results showed that the MICs of three drugs could be reduced upon *clpP1*‐KD induction (Table 1). Building on these observations, together with the proteomic data, we pinpointed two synergistic combinations: GDI‐5755–ETH and GDI‐5755–MPM. The synergistic effects were also confirmed with the Mtb H37Ra strain (data not shown). One limitation to note is that the proteomics was not optimized to detect membrane‐embedded proteins, including the EMB targets^47^, EmbB and EmbA, thus potentially leading to inaccuracies in quantifying the arabinosyltransferase levels. Subsequently, with the checkerboard assay, we found that the combination of GDI‐5755 and EMB showed additive effects in BCG. Another avenue not explored in this study was the combination of GDI‐5755 with PZA, despite proteomic evidence indicating a significant reduction in the levels of PanD, the enzyme targeted by PZA, in GDI‐5755‐treated cells. Future investigations will ascertain the impact of this combination, making use of a more potent ClpP inhibitor with improved physicochemical properties and testing the efficacy against Mtb cultures in a pH‐adjusted medium. The integrative approach of combining proteomic analyses, chemical–genetic screens, and checkerboard assays offers robust validation for our conclusions. In line with the experiences shared by other researchers^48^, ^49^, ^50^, ^51^, we advocate for the use of chemical–genetic interaction as an invaluable methodology in the drug discovery process. Such approaches not only provide important insights into the druggability of targets but also enhance our understanding of their biological functions. This knowledge will be instrumental in devising combination therapies that can effectively treat complex diseases such as tuberculosis.

## MATERIALS AND METHODS

### Bacterial strains, plasmids, primers, culture conditions, and compounds

*Mycobacterium* strains used in this study are BCG Str. Pasteur 1173P2 and *M. tuberculosis* H37Rv ATCC 27294. They were grown in liquid Middlebrook 7H9 medium (Difco) supplemented with 0.5% glycerol, 10% oleic acid–albumin–dextrose–catalase (OADC), and 0.05% tyloxapol. *Escherichia coli* was grown in LB media at 37°C and shaking at 180 rpm, and when necessary, the cultural medium or plate was supplemented with kanamycin at 50 µg/ml for *E. coli* and 25 µg/ml for mycobacteria. The plasmid PLJR965 (Addgene #115163) used for the construction of the CRISPRi strain was purchased. Details of strains, plasmids, and DNA primers are provided in Table S1. Synthesis information of GDI‐5755, 5756, and 5757 is provided in the Supporting Information section. The compounds were dissolved in dimethylsulfoxide (DMSO) to prepare a 20 mM stock solution. The antibiotics and other reagents were purchased from MCE. Activator dipeptide Bz‐HT (*N*‐benzoyl His‐Thr aldehyde) and fluorescent tripeptide substrate *N*‐acetyl Pro‐Lys‐Met‐aminomethylcoumarin (Ac‐PKM‐amc) were synthesized in GenScript.

### Protein expression, purification, and high‐throughput screening

The high‐throughput screening assay was developed based on a previously described method, with some modifications^13^, ^52^. The genes encoding N‐terminally truncated ClpP1(7‐200) and ClpP2(16‐214) fused with a His_6_‐tag at C‐terminus were, respectively, cloned in the pET21b plasmid and the plasmids were transformed into *E. coli* BL21(DE3) cells for expression. The truncated ClpP1 and ClpP2 were purified with an immobilized metal‐affinity chromatography (IMAC), followed by Hitrap Q HP anion exchange chromatography. A 0–0.5 mM NaCl gradient eluted fraction containing pure ClpP1 and ClpP2 proteins was analyzed and confirmed with SDS‐PAGE analysis. The fractions were pooled, concentrated to ~8 mg/ml, and stored at −80°C. 200‐nl of the library compound was dispensed into each well of a 384‐well plate (Thermo 262260). Equal molar ratios of ClpP1 and ClpP2 proteins and the activator dipeptide were mixed in a tube for 45 min. 20 µl of the mixture was dispensed into each well of the 384‐well plate to mix. Then, the other components of the reaction including substrate Ac‐PKM‐amc and buffer were added to a well to make up a total volume of 40 µl. The final concentrations in each well are 100 nM ClpP1, 100 nM ClpP2, 100 μM Ac‐PKM‐amc, and 0.5 mM Bz‐HT in 30 mM PIPES buffer (pH 7.5) with 200 mM NaCl and 0.005% Tween20. The increase in fluorescence was immediately recorded on a plate reader Spectramax M5 (Molecular Devices) with the temperature setting at 37°C and excitation and emission at 380/460 nm. Bortezomib and DMSO were utilized as positive and negative controls, respectively. The Z’ factor of all screening plates was ~0.8. The inhibition rate is calculated using Equation (1). *F* stands for the actual reading of each well.

(1) $$\text{Inhibition rate (\%)}=\frac{{\text{F}}_{\text{DMSO}}-{\text{F}}_{\text{compound}}}{{\text{F}}_{\text{DMSO}}-{\text{F}}_{\text{bortezomib}}}\times 100\%$$

### Peptidase degradation assay and IC_50_ determination

The peptidase activity was measured by recording the fluorescence resulting from the degradation of 10 μM substrate Ac‐PKM‐amc by 50 nM ClpP1P2 hetero‐tetradecamers in the presence of 0.5 mM Bz‐HT, in a buffer of 30 mM PIPES (pH7.6), 200 mM NaCl, and 0.005% Tween 20, in a final volume of 40 µl for at least 1 h at 37°C. The inhibitory activity of a compound leads to an increase in fluorescence with increasing compound concentration. To determine the IC_50_ of inhibitory activity, the serially diluted concentrations of GDI‐5755 and fluorescence readouts were fitted into the log (inhibitor) versus response—variable slope (four parameters) in GraphPad Prism 10.

### Jump dilution experiment and determination of the *k*_obs_/[I]

10 µM ClpP1P2 was pre‐incubated with 10 µM GDI‐5755 or DMSO solvent for 60 min at room temperature. To induce the formation of the [EI] complex, 0.5 mM Bz‐HT was also added. The mixture was diluted 100‐, 200‐, or 400‐fold to a 50 µl final volume with the peptidase reaction buffer and 20 μM substrate to start the jump dilution experiment. Fluorescence was recorded every 5 min continuously over a 220‐min course. To determine the *k*_obs_ of the competitive inhibitor, the compound was dispended into a black 384‐well plate in a twofold concentration gradient from 20 µM to 0.5 µM. Without pre‐incubation, the other components (50 nM ClpP1P2, 200 µM substrate, and 0.5 mM Bz‐HT in the PIPES buffer as above) were added like in the peptidase degradation assay. The progress was monitored by continuously recording the fluorescence readouts. The fluorescence vs reaction time in seconds was fitted into Equation (2) using GraphPad Prism 10.

(2) $$[P]=\nu_{\text{s}}t+\frac{\nu_{\text{i}}-\nu_{\text{s}}}{k_{\text{obs}}}\left[1-\text{exp}\left(-k_{\text{obs}}t\right)\right]$$

where *P* represents the product concentration, *v*_s_ is the steady‐state reaction rate (0 in this case), *v*_i_ is the initial rate, *t* is a time point, and *k*_obs_ is the first‐order rate constant for onset of inhibition. The *k*_obs_ values obtained were plotted against the inhibitor concentration and fitted into linear regression to yield *k*_obs_/[I] values as the slope, indicating the inhibition potency of the compound.

### Intact protein MS

10 μM of the ClpP1(7‐200) ClpP2(16‐214) enzyme complex was incubated with DMSO (as negative control) and 100 μM GDI‐5755 separately in 30 mM PIPES buffer only at room temperature in a final reaction volume of 40 μl. After 1 h of incubation, the samples were sent to a service lab at Peking University for a Matrix‐Assisted Laser Desorption/Ionization (MALDI) Time‐of‐Flight (TOF) MS analysis.

### Construction and confirmation of *clpP* CRISPRi strains

The CRISPRi plasmids targeting the non‐template strand of *clpP1* and *clpP2* genes were constructed following the previously described methods^26^. Briefly, to clone the sgRNA targeting sequences into the sgRNA scaffold, four pairs of complementary primers were synthesized as oligos with GGGA and AAAC overhangs, respectively, with one pair designed as the scrambled sgRNA (Table S2). These primers were annealed and cloned into the BsmB1‐digested pLJR965 backbone using T4 DNA ligase (400U/µl, NEB) at 20°C for 20 min. The resulting sgRNA‐containing CRISPRi plasmids were then transformed into DH5a‐competent cells and verified by Sanger sequencing. Confirmed sgRNA CRISPRi expression plasmids were subsequently electroporated into wild‐type BCG. Stable transformants with the plasmid integrated into the L5 site in the genome were confirmed by PCR analysis with the primers listed in Table S3. The knockdown strains are designated GH189 (c*lpP1*‐KD) and GH200 (control). The transcriptional level of *clpP1* and *clpP2* was analyzed using gene‐specific primers via quantitative RT‐PCR.

### Extraction of the bacterial RNA

Briefly, the BCG and its deviated strains carrying pLJR965 with either sgRNA targeting the *clpP1* gene or scrambled sgRNA were grown in 7H9 medium supplemented with 20 µg/ml kanamycin to an optical density (OD_600_) of 0.5. The cultures were then diluted to an OD_600_ of 0.005 and induced with or without 4 ng/ml ATc at 37°C with shaking for 5 days. The cultures were harvested by centrifugation at 4000 rpm for 10 min, the pellets were washed with PBS (pH7.4) and resuspended in 2 ml of RNAPprotect Bacteria Reagent (Qiagen), and incubated at room temperature for 5 min. Following incubation, the cells were collected by centrifugation, then resuspended in 350 µl of lysis buffer supplemented with 3.5 µl of β‐mercaptoethanol, and mechanically disrupted with 0.1 mm Zirconia beads in a Precellys Evolution Touch homogenizer (Bertin Technologies) at 7200 rpm for four cycles, with a 4‐min ice interval between cycles. Following centrifugation at 15,000 rpm for 10 min at 4°C, the supernatant was transferred to a new 1.5 ml tube. RNA extraction was then performed according to the GeneJET RNA Purification Kit (Thermo Scientific) protocol. The Genomic DNA contamination was removed using the On‐Column DNase I Digestion Set, and RNA was finally dissolved in 50 µl of RNase‐free water. The quantity and purity of the RNA were assessed by 1.5% agarose gel electrophoresis, and the absence of genomic DNA contamination was confirmed by PCR on the *rpoB* and 16 s RNA with the RNA samples as templates.

### Quantitative reverse‐transcription PCR (qRT‐PCR)

The purified RNA (300 ng) was used to synthesize cDNA by reverse transcription using the First Strand cDNA Synthesis Kit for RT‐PCR (AMV). The qRT‐PCR analysis was performed using the QuantStudio^TM^ 7 Flex Real‐time PCR system (Thermo Fisher Scientific) with TB Green® Premix Ex Taq™ II (Tli RNaseH Plus) (Takara) according to the manufacturer's instructions. Primers for qRT‐PCR are listed in Table S4. Two biological replicates were carried out for the quantification of the genes. The RNA polymerase subunit *rpoB* was used as the internal control. Data were analyzed using the comparative Ct method and the relative fold change was calculated as $2^{-\Delta \Delta C_{T}}$. Statistical analysis was performed using one‐way ANOVA and Dunnett's multiple comparisons test.

### MIC and MIC‐shifting assays with hit compounds

MIC for each compound was determined using the resazurin reduction microplate assay (REMA) as previously described^53^. Briefly, a compound with an original concentration of 360 µM was used to prepare a twofold dilution, and the prepared *M. bovis* bacterial suspension was added to each well to obtain an estimated 2 × 10^5^ CFU/ml with a total volume of 200 µl. The well only containing 0.8% solvent DMSO or isoniazid in the 7H9 medium was used as a control. After 7 days of incubation, 50 µl of a freshly prepared 1:1 mixture of alamarBlue Reagent (Invitrogen) and 10% Tween 80 was added to each well and incubated at 37°C in 5% CO_2_ for 24 h to allow the cells to convert resazurin into resorufin. Measuring the fluorescence at excitation of 540 nm and emission of 590 nm, a MIC value referred to in this work with BCG is defined as the lowest concentration causing a 50% inhibition of fluorescence relative to the untreated bacterial control, except for the specific MICs measured with Mtb H37R_v_. To verify that a compound of interest is on target, the conditional mutant *clpP1*‐KD (GH189) and the control strain expressing scrambled sgRNA were cultured in 7H9 medium supplemented with 10% OADC, 0.05% tyloxapol, and 20 µg/ml kanamycin until the optical density reached 0.4−0.6 (OD_600_). A 9‐point, twofold dilution gradient of the compound was prepared and dispensed into each well of a 96‐well plate containing 100 µl of the 7H9‐OADC‐Tyloxapol medium. The cultured *clpP1*‐KD strain or derivates was diluted in the 7H9‐OADC‐Tyloxapol medium at a ratio of 1:100 with various concentrations of ATc induction (ATc = 0, 2, 4, 6 ng/ml). Then, 100 µl of the *clpP1*‐KD strain was inoculated with various concentrations of ATc into the corresponding wells of the 96‐well plates containing the twofold diluted compound and control well and the plates were incubated at 37°C for 7 days. The MIC of the *clpP1‐KD* strain at various concentrations of ATc was calculated as described above. All data are representative of three independent experiments. MIC was calculated by curve fitting the data of the relative growth versus drug concentration to a model named “log (inhibitor) versus normalized response − Variable slope” in GraphPad Prism 9, and a graph was then generated.

### Preparation of mouse polyclonal antisera and the Western blot analysis

For immunization, 20 μg of purified ClpP2 protein was mixed with Freund's incomplete adjuvant at a ratio of 1:1 (v/v) and used for the primary immunization of ten mice by a subcutaneous multi‐point injection. The polyclonal antiserum against ClpP2 was raised by three immunizations, and antiserum was collected after the third immunization. To validate the *clpP1*‐KD strain, immunoblotting was used to assess whether the ClpP2 protein level decreases in the *clpP1*‐KD strain in the presence of ATc. The *M. bovis clpP1*‐KD cultures were synchronized in growth before the addition of various concentrations of ATc. For each time point, 10 ml of culture was harvested (3600*g*, 10 min) and resuspended in 500 µl of lysis buffer, 50 mM HEPES, and 150 mM pH 7.4 NaCl, supplemented with the cOmplete^TM^ (Roche) EDTA‐free protease inhibitor cocktail tablet. The sample was lysed by bead beating in the tube for four cycles at 7200 rpm (45 s intervals on ice) using the Precellys Evolution Touch homogenizer (Bertin Technologies). The cell lysates were centrifugated at 20,000 rpm for 10 min, and the supernatant was transferred to a new 1.5 ml EP tube. Subsequently, 30 µl of the lysate was mixed with 4× LDS sample buffer (Thermo Scientific) and heated for 10 min at 99°C. Samples were separated on a 4–12% bis‐tris polyacrylamide gel and transferred onto a PVDF membrane using the iBlot™ 2 Transfer Stacks and iBlot™ 2 Gel Transfer Device (Invitrogen). The membrane was incubated with a 1:1000 dilution of polyclonal antiserum against ClpP2 of *M. bovis*, followed by incubation with a 1:10000 dilution of anti‐mouse IgG antibody as the secondary antibody and RpoB as the inner control. The blot was detected using pierce ECL Western reagent (Thermo Fisher Scientific).

### Preparation of whole‐cell lysates (WCLs) and protein extraction

The *M. bovis* cultures were synchronized in growth to the logarithmic phase (OD_600_ ~ 0.1) before treatment with or without 2×MIC concentrations of GDI‐5755 and incubated at 37°C in 5% CO_2_. 10 ml of culture was harvested after 2 and 3 days of treatment via centrifugation at 3600*g* for 10 min, and then washed twice with PBS (pH 7.4). Subsequently, the cells were resuspended in 500 µl of PBS supplemented with the protease inhibitor tablet and lysed by zirconia beads in the tube for four cycles at 7200 rpm at 45 s intervals on ice using the Precellys Evolution Touch homogenizer (Bertin Technologies). The cell lysates were centrifugated at 20,000 rpm for 10 min, and the supernatant was transferred to a new 1.5 ml EP tube. The supernatant was quantified using the Pierce™ BCA Protein Assay Kits before being flash‐frozen and stored at −80°C.

To extract the total protein from the WCLs, a 1× protease inhibitor cocktail containing EDTA (diethylamine tetraacetic acid) was added. The supernatant was obtained through ultrasonic crushing and cracking, followed by centrifugation at 25,000*g* and 4°C for 15 min. Next, DTT (dithiothreitol) was added to a final concentration of 10 mM and incubated at 37°C for 30 min. Subsequently, IAM (iodoacetamide) was added at a final concentration of 55 mM and the sample was placed in the dark for 45 min. Afterward, the sample was treated with five times the volume of pre‐cooled acetone, followed by incubation at −20°C for 2 h, and centrifugation at 25,000*g* and 4°C for 15 min. The supernatant was discarded and the remaining precipitate was air‐dried. SDS‐free protein cracking solution was added, and an automatic grinding instrument was used to promote protein dissolution. Finally, the sample was centrifuged at 25,000*g* and 4°C for 15 min, and the supernatant was quantified using the Pierce™ BCA Protein Assay Kits before assessing the quality of the prepared samples by SDS‐PAGE. For processing the protein samples for proteomics and peptide identification, etc., please refer to additional methods provided in the supplementary.

### Checkerboard assay

To confirm whether the ClpP‐targeting compound shows synergistic, antagonistic, or additive effects against Mycobacteria, a checkerboard assay was carried out using REMA. Anti‐Mtb drug (ETH and MPM) concentrations ranging from 8× MIC to 0 were prepared in 96‐well plates by twofold serial dilution, and this serially diluted drug interacted with the ClpP‐targeting compound GDI‐5755 at various concentrations by twofold serial dilution. Each well contained 2 × 10^5^ CFU/ml *M. bovis* with a total volume of 200 µl. Each plate was incubated for 7 days at 37°C before adding resazurin. The fluorescence was measured at excitation of 540 nm and emission of 590 nm. The MIC is defined as the lowest concentration that results in a 90% reduction in fluorescence compared to untreated controls. The FICI was calculated based on the sum of the FIC of each drug in the indicated well, as follows: FICI = MIC_A in the presence of B_ /MIC_A alone_ + MIC_B in the presence of A_ /MIC_B alone._ FICI ≤ 0.5 indicates synergism; 0.5 < FICI ≤ 1 indicates an additive effect; 1 < FICI < 2, indicates no interaction; and FICI ≥ 2 indicates antagonism.

## AUTHOR CONTRIBUTIONS

**Genhui Xiao**: Data curation (equal); formal analysis (lead); investigation (lead); methodology (equal); validation (equal); and writing—original draft (equal). **Yumeng Cui**: Data curation (equal); formal analysis (equal); investigation (equal); validation (equal); and writing—original draft (equal). **Liangliang Zhou**: Data curation (equal); formal analysis (equal); methodology (equal); validation (equal); and writing—original draft (equal). **Chuya Niu**: Data curation (equal); investigation (equal); and methodology (equal). **Bing Wang**: Data curation (equal) and formal analysis (equal). **Jinglan Wang**: data curation (equal) and methodology (equal). **Shaoyang Zhou**: Data curation (equal). **Miaomiao Pan**: Data curation (equal). **Chi Kin Chan**: Investigation (equal) and resources (equal). **Yan Xia**: Supervision (equal) and validation (equal). **Lan Xu**: Resources (equal); supervision (equal); and validation (equal). **Yu Lu**: Project administration (equal); resources (equal); and supervision (equal). **Shawn Chen**: Conceptualization (lead); data curation (lead); formal analysis (equal); funding acquisition (lead); investigation (lead); methodology (supporting); project administration (lead); resources (equal); supervision (lead); validation (lead); writing—original draft (equal); and writing—review and editing (lead).

## ETHICS STATEMENT

No animals or humans were involved in this study.

## CONFLICT OF INTERESTS

The authors declare no conflicts of interests.

## Supporting information

Supporting information.

Supporting information.

Supporting information.

## Data Availability

All data generated or analyzed in this study are presented within this article.
