# Supplementary material for: Identification of a phenyl ester covalent inhibitor of caseinolytic protease and analysis of the ClpP1P2 inhibition in mycobacteria
Source: mLife. 2025 Apr 15;4(2):155–68. doi: 10.1002/mlf2.12169 (PMC12042115; doi:10.1002/mlf2.12169)
Supplement: Supplementary file 1 — Supporting information. [file MLF2-4-155-s001.pdf]

Supplementary Tables

Table S1. Bacterial strains and plasmids used in this study**.**

Strains	Description	Reference or source
M. bovis	*Mycobacterium bovis* (strain BCG/ Pasteur 1173P2)	L. Lyu in Fudan University
GH189	*M. bovis* BCG derivative, BCG was transformed with the pLJR965 expressing the sgRNA to target the clpP1 gene	This study
GH200	*M. bovis* BCG derivative, BCG was transformed with the pLJR965 containing the scramble sgRNA as negative control	This study
DH5a	F- endA1 glnV44 thi-1 recA1 relA1 gyrA96 deoR nupG φ80lacZΔM15 Δ (lacZYA-argF) U169 hsdR17 (rK^- mK^+) $λ^-$	TransGen Biotech
BL21	F-dcm ompT hsdS(r_B- m_B-) gal $[malB^+]_{K\text{-}12}(λ^S)$	TransGen Biotech
**Plasmids**		
pLJR965	Sth1 dCas9 TetR and KanR L5 Int attP for *M. tuberculosis*	Addgene #115163
pET21b	*E. coli* protein expression vector, amp^r	Novagen

Table S2. Primers synthesized to clone the sgRNAs into pLJR965 for CRISPR-mediated knocking down target gene in *M. bovis* BCG as previously described for Mtb.

Target gene	Name of oligos	Forward primer (5'-3')	Reverse primer (5'-3')
clpP1	sgRNA1	GGGAGCTGGCGTCTTCGGCGGCCAGCA	AAACTGCTGGCCGCCGAAGACGCCAGC
	sgRNA2	GGGAATCTCGTCGTTCACCTCCGAGCC	AAACGGCTCGGAGGTGAACGACGAGAT
control	Scramble sgRNA	GGGAGAGACGATTAATGCGTCTCG	AAACCGAGACGCATTAATCGTCTC

Table S3. Primers for the Identification of the constructed CRISPRi-strains by PCR

Primers	Description	Reference or source
Pr2050	5'-CACCAACTGGTCCACCTAC-3'	This study
Pr2051	5'-CCTGCGTTATCCCCTGAT-3'	This study
Pr2058	5'-ACGACCGAGCGCAACGCGTG-3'	This study
Pr2068	5'-GTAGGCCCGCTGGAAATCCG-3'	This study
Pr2077	5'-GAGCCAGGCTTCGGCGTCC-3'	This study
Pr2069	5'-GCCGCGCCGACCTCGTTGC-3'	This study

Table S4. Primers were synthesized to quantify the mRNA expression level of target genes by qRT-PCR in *M. bovis* BCG at the integrated DNA Technologies website (https://sg.idtdna.com).

Target gene	Gene name	Forward primer (5'-3')	Reverse primer (5'-3')
BCG_2482c	*tig*	CGAGCTTGAGCCGGATTT	CGGGCTTCGAGTAGTTTGG
BCG_2479	*jefA*	CTCTTCGTCGTGAGCTCTATTG	GATGAACGCCGCTCCTAAA
BCG_2481c	*clpP1*	CGCCGTGATCAAGAAAGAAAG	AAACCGTATTCCAGGGCTTC
BCG_2480c	*clpP2*	GCACCCTGATGGAAACCA	GATGATGCCGTAGTCCTTAGC
BCG_0716	*rpoB*	CGGTCGCTATAAGGTCAACAA	TGCAAGCGGACCAGATATTC

Table S5. The orthologous proteins that downregulated in the GDI5755-treated BCG strain were commonly down-regulated in Mth after depletion of clpP1P2, as analyzed by quantitative proteomics in this and previous reports (Raju et al. Plos Pathogen 2014, 10(3): e1003994).

No.	Protein_ID	BCG locus	H37Rv locus	Description	Mean_Ratio GDI5755_3d: DMSO_3d	*Mean_Ratio ClpP1 Mutant: WT	**Essentiality
1	A0A0H3M1L1	BCG_0495c	Rv0456c	Enoyl-CoA hydratase echA2	0.5	0.5	No
2	A1KMY2	BCG_3009c	Rv2988c	3-isopropylmalate dehydratase large subunit	0.6	0.6	Growth Defect
3	A0A0H3M7N7	BCG_2886c	Rv2864c	Possible penicillin-binding lipoprotein	0.6	0.6	No
4	A0A0H3MBE4	BCG_1962	Rv1923	Probable lipase lipD	0.6	0.8	Growth Advantage
5	A0A0H3M2R2	BCG_0692c	Rv0643c	Methoxy mycolic acid synthase 3 mmaA3	0.6	0.5	Growth Advantage
6	P80069	BCG_1896	Rv1860	Alanine and proline-rich secreted protein Apa	0.6	0.6	No
7	A0A0H3M8B1	BCG_0546c	Rv0503c	Cyclopropane-fatty-acyl-phospholipid synthase 2 cmaA2	0.7	0.7	Growth Advantage
8	A0A0H3M694	BCG_1579c	Rv1527c	Mycocerosic acid synthase-like polyketide synthase	0.7	0.6	No
9	A0A0H3M762	BCG_1922c	Rv1885c	Chorismate mutase	0.7	0.8	Growth Advantage
10	A1KJU9	BCG_1923c	Rv1886c	Mycolyltransferase Ag85B	0.7	0.8	Growth Advantage
11	A0A0H3M3P2	BCG_1048	Rv0993	UTP--glucose-1-phosphate uridylyltransferase	0.7	0.8	Essential
12	A0A0H3M488	BCG_1619c	Rv1566c	Possible inv protein	0.7	0.2	Growth Advantage
13	A0A0H3M0U8	BCG_0210	Rv0173	MCE-family lipoprotein lprK	0.7	0.6	No
14	A0A0H3MFS1	BCG_2550	Rv2529	ERCC4 domain-containing protein	0.7	0.8	No
15	A0A0H3MIY3	BCG_3772	Rv3712	Lipid II isoglutaminyl synthase subunit MurT	0.7	0.7	Essential
16	A0A0H3M1I8	BCG_0209	Rv0172	MCE-family protein mce1D	0.7	0.6	No
17	A0A0H3M233	BCG_0693c	Rv0644c	Methoxy mycolic acid synthase 2 mmaA2	0.7	0.7	Growth Advantage
18	A0A0H3M2P5	BCG_0207	Rv0170	MCE-family protein mce1B	0.7	0.8	No
19	A0A0H3M7F0	BCG_0211	Rv0174	MCE-family protein mce1F	0.7	0.6	No
20	A0A0H3M760	BCG_0066	Rv0035	Fatty-acid-coa ligase fadD34	0.7	0.5	No
21	A0A0H3MC88	BCG_2317	Rv2301	Cutinase	0.7	0.7	No
22	A0A0H3MC34	BCG_2260	Rv2243	Malonyl CoA-acyl carrier protein transacylase fabD	0.7	0.4	Essential
23	A0A0H3MC70	BCG_1203c	Rv1141c	Probable enoyl-CoA hydratase echA11	0.7	0.8	No
24	A0A0H3M7W0	BCG_2977	Rv2956	Methyltransferase FkbM domain-containing protein	0.7	0.7	No
25	A0A0H3M7Y1	BCG_0442	Rv0404	Probable fatty-acid-CoA ligase fadD30	0.7	0.6	No
26	A0A0H3MDI1	BCG_3731c	Rv1665	Possible chalcone synthase pks11	0.7	0.7	No

No.	Protein_ID	BCG locus	H37Rv locus	Description	Mean_Ratio GDI5755_3d: DMSO_3d	*Mean_Ratio ClpP1 Mutant: WT	**Essentiality
27	A0A0H3M1V2	BCG_0591c	Rv0547c	Possible oxidoreductase	0.8	0.7	No
28	A0A0H3M5L3	BCG_1311c	Rv1251c	Uncharacterized protein	0.8	0.5	No
29	A0A0H3M848	BCG_3111	Rv3086	alcohol dehydrogenase adhD	0.8	0.8	No
30	A0A0H3M782	BCG_0101c	Rv0070c	Serine hydroxy methyltransferase	0.8	0.6	No
31	A0A0H3M8J9	BCG_3296	Rv3267	LytR family transcriptional regulator	0.8	0.6	No
32	A0A0H3MII7	BCG_3658	Rv3593	Probable conserved lipoprotein lpqF	0.8	0.8	No
33	A0A0H3M4W9	BCG_1534	Rv1473	Macrolide-transport ATP-binding protein ABC transporter	0.8	0.8	No
34	A0A0H3MDH4	BCG_1699	Rv1660	Possible chalcone synthase pks10	0.8	0.7	No
35	A0A0G2Q9F4	BCG_0001	Rv0001	Chromosomal replication initiator protein DnaA	0.8	0.7	Essential
36	A0A0H3MAW4	BCG_3375	Rv3310	Possible acid phosphatase	0.8	0.4	No
37	A1KQF4	BCG_3882c	Rv3820c	Trehalose-2-sulfate acyltransferase PapA2	0.8	0.7	No
38	A0A0H3M9Y2	BCG_0208	Rv0171	MCE-family protein mce1C	0.8	0.7	No
39	A0A0H3M3S6	BCG_0547c	Rv0504c	UPF0336 protein	0.8	0.7	No
40	A0A0H3M7D3	BCG_2546c	Rv2525c	Putative peptidoglycan hydrolase	0.8	0.4	No
41	A0A0H3M5U3	BCG_2254	Rv2237	ER-bound oxygenase mpaB	0.8	0.8	No
42	A1KQD8	BCG_3866c	Rv3804c	Mycolyltransferase Ag85A	0.8	0.7	No
43	A0A0H3M5Y7	BCG_2310	Rv2294	Probable aminotransferase	0.8	0.8	No

* Data from Raju et al. Plos Pathogen 2014, 10(3): e1003994

** As marked in the portal, https://pebble.rockefeller.edu/

Table S6. The orthologous proteins that accumulated (upregulated) in the GDI5755-treated BCG strain were commonly up-regulated in Mtb after depletion of clpP1P2, as analyzed by quantitative proteomics in this and previous reports (Raju et al. PLos Pathogen 2014, 10(3): e1003994).

No.	Protein_ID	BCG locus	H37Rv locus	Description	Mean_Ratio GDI5755_3d: DMSO_3d	*Mean_Ratio ClpP1 Mutant: WT	**Essentiality
1	A0A0H3MA45	BCG_0289c	Rv0251c	Heat stress-induced ribosome-binding protein A, acr2	3.9	75.9	No
2	A0A0H3M8B9	BCG_3185c	Rv3161c	Dioxygenase	2.2	1.7	No
3	A0A0H3MFK9	BCG_2486c	Rv2466c	DSBA-like thioredoxin domain-containing protein	1.7	2.4	No
4	A0A0H3MB64	BCG_3476	Rv3406	Probable dioxygenase	1.6	3.3	No
5	A1KFH2	BCG_0389	Rv0350	Chaperone protein DnaK	1.5	2.2	Essential
6	A0A0H3M380	BCG_0391	Rv0352	Chaperone protein DnaJ	1.5	2.0	No
7	A0A0H3M0N8	BCG_0130	Rv0097	decarboxylase	1.5	1.5	No
8	A0A0H3MB87	BCG_1890c	Rv1854c	NADH dehydrogenase ndh	1.5	4.2	Growth Defect
9	A0A0H3M7W9	BCG_0422c	Rv0384c	Chaperone protein ClpB	1.5	3.9	Growth Defect
10	A0A0H3MBY6	BCG_3824c	Rv3765c	Two-component transcriptional regulator TcrX	1.4	2.1	No
11	A0A0H3M2T8	BCG_0726c	Rv0677c	Possible conserved membrane protein mmpS5	1.4	2.1	No
12	A0A0H3MBZ0	BCG_1101c	Rv1043c	Serine protease	1.4	1.4	Growth Advantage
13	A0A0H3M5K5	BCG_1787	Rv1748	DUF4129 domain-containing protein	1.4	1.7	No
14	A0A0H3MBE7	BCG_3590	Rv3526	Rieske-type oxygenase	1.4	2.1	No
15	A0A0H3M896	BCG_2934c	Rv2913c	Possible D-amino acid aminohydrolase	1.4	1.5	No
16	A0A0H3MD16	BCG_2656	Rv2629	Uncharacterized protein	1.4	3.4	No
17	A0A0H3M9C3	BCG_2721c	Rv2708c	DUF3039 domain-containing protein	1.4	1.8	Growth Advantage
18	A0A0H3M8U1	BCG_3153c	Rv3130c	Diacylglycerol O-acyltransferase	1.4	2.4	No
19	A0A0H3MBR5	BCG_1028c	Rv0974c	Probable acetyl-/propionyl-coa carboxylase, accD2	1.4	1.3	No
20	A1KFH3	BCG_0390	Rv0351	Protein GrpE/HSP-70 cofactor	1.4	1.7	Essential
21	A0A0H3MAY5	BCG_0774	Rv0724	Possible protease IV sppA (Endopeptidase IV)	1.4	2.0	No
22	A0A0H3MA52	BCG_3881	Rv3819	Uncharacterized protein	1.4	1.3	No
23	A0A0H3M270	BCG_0463c	Rv0424c	Uncharacterized protein	1.4	1.7	No
24	A0A0H3M7S6	BCG_2723	Rv2710	RNA polymerase sigma factor, sigB	1.3	10.8	No
25	A0A0H3M3I9	BCG_1334	Rv1275	Lipoprotein lprC	1.3	1.6	No
26	A0A0H3M955	BCG_0993	Rv0939	Bifunctional 2-hydroxyhepta-2,4-diene-1,7-dioate isomerase	1.3	2.3	No

* Data from Raju et al. Plos Pathogen 2014, 10(3): e1003994.   ** As marked in the portal, https://pebble.rockefeller.edu/

No.	Protein_ID	BCG locus	H37Rv locus	Description	Mean_Ratio GDI5755_3d: DMSO_3d	*Mean_Ratio ClpP1 Mutant: WT	**Essentiality
27	A0A0H3MA44	BCG_3709	Rv3651	Rv3651-like N-terminal domain-containing protein	1.3	1.4	No
28	A0A0H3MAB2	BCG_1492	Rv1431	Conserved membrane protein	1.3	3.8	No
29	A0A0H3M3I2	BCG_1324	Rv1265	Uncharacterized protein	1.3	2.3	No
30	A0A0H3MJD0	BCG_3905c	Rv3842c	Glycerol phosphoryl diester phosphodiesterase glpQ1	1.3	1.4	Growth Advantage
31	A1KIS1	BCG_1543	Rv1481	UPF0353 protein	1.3	1.4	Essential
32	A0A0H3MAA4	BCG_0347c	Rv0307c	Acetoacetate decarboxylase	1.3	2.9	No
33	A0A0H3ME28	BCG_3165c	Rv3142c	Uncharacterized protein	1.3	1.5	No
34	A0A0H3M5W7	BCG_1937	Rv1898	Thiamine-binding protein-containing protein	1.3	1.8	No
35	A0A0H3M638	BCG_1532	Rv1471	Thioredoxin	1.3	4.5	No
36	A1KKK9	BCG_2183c	Rv2166c	Transcriptional regulator MraZ	1.3	1.5	No
37	A1KIJ9	BCG_1471c	Rv1410c	Probable triacylglyceride transporter	1.3	1.4	No
38	A0A0H3MCR6	BCG_2516c	Rv2496c	pyruvate dehydrogenase E1 component pdhB	1.3	3.1	No
39	A0A0H3M9B9	BCG_3583	Rv3519	Acetoacetate decarboxylase	1.3	1.6	No
40	A0A0H3MCR5	BCG_1438c	Rv1377c	Transferase	1.3	1.3	No
41	A0A0H3MA04	BCG_3674c	Rv3610c	ATP-dependent zinc metalloprotease FtsH	1.3	1.5	No
42	A0A0H3M628	BCG_2351c	Rv2330c	Probable lipoprotein lppP	1.3	1.9	No
43	A0A0H3M5J1	BCG_1283	Rv1223	Probable serine protease htrA	1.3	1.3	Essential
44	A0A0H3M8Y9	BCG_0909	Rv0857	Cyclase	1.3	2.0	No
45	A0A0H3MEB3	BCG_1984	Rv1148c	HNH nuclease domain-containing protein	1.3	4.1	No
46	A0A0H3MF17	BCG_2241c	Rv2224c	Serine protease Hip1	1.3	1.4	Growth Advantage
47	A0A0H3MD01	BCG_1523	Rv1462	Fe-S cluster assembly protein SufD	1.3	1.3	Essential
48	A0A0H3MDS7	BCG_3028	Rv3006	conserved lipoprotein lppZ	1.3	1.6	Essential
49	A0A0H3M4G0	BCG_1333	Rv1274	Lipoprotein lprB	1.3	1.4	No
50	A0A0H3M392	BCG_1189c	Rv1128c	HNH nuclease domain-containing protein	1.3	6.9	No
51	A0A0H3M899	BCG_3164	Rv3141	Probable NADPH quinone oxidoreductase fadB4	1.2	1.6	No
52	A1KHB8	BCG_1037	Rv0982	Two-component sensor kinase MprB	1.2	1.6	Essential
53	A1KNP2	BCG_3269c	Rv3240c	Protein translocase subunit SecA 1	1.2	1.5	Essential

Supplementary Figures

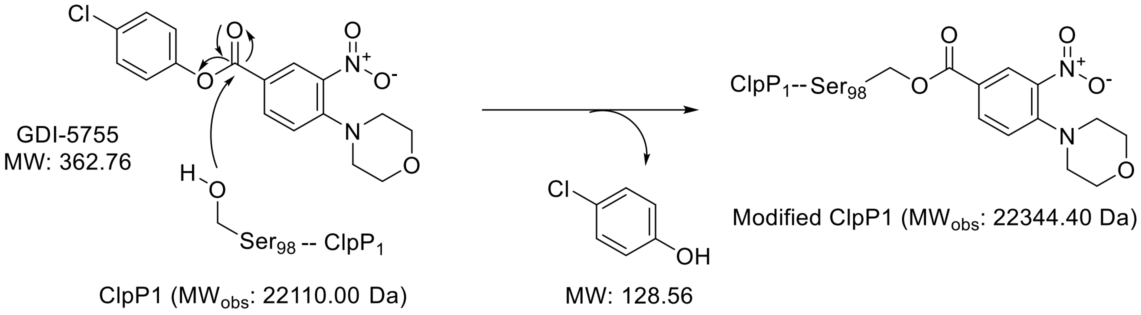

Figure S1. Reaction scheme for the GDI-5755 modification of Ser98 in the ClpP1.

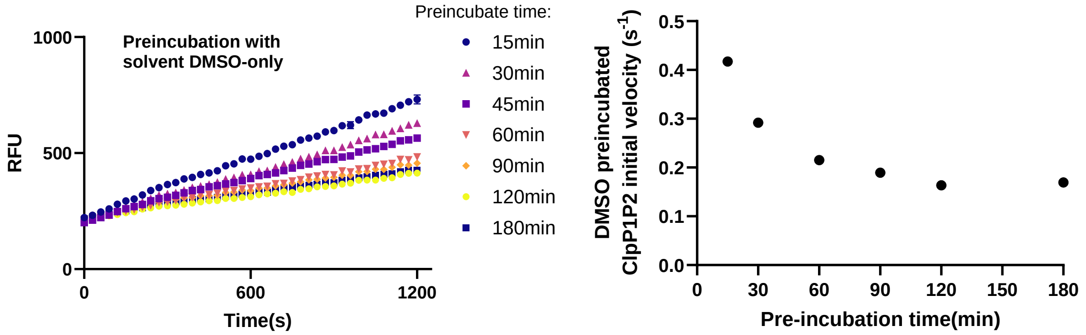

Figure S2. Progression (left) and initial velocity V_i (right) of ClpP1P2 peptidase reaction preincubated with solvent for varying amounts of time. No GDI-5755 inhibitor was included in the preincubation mixture. Instead, the same amount of solvent DMSO (less than 2% of total volume) was added to the preincubation.

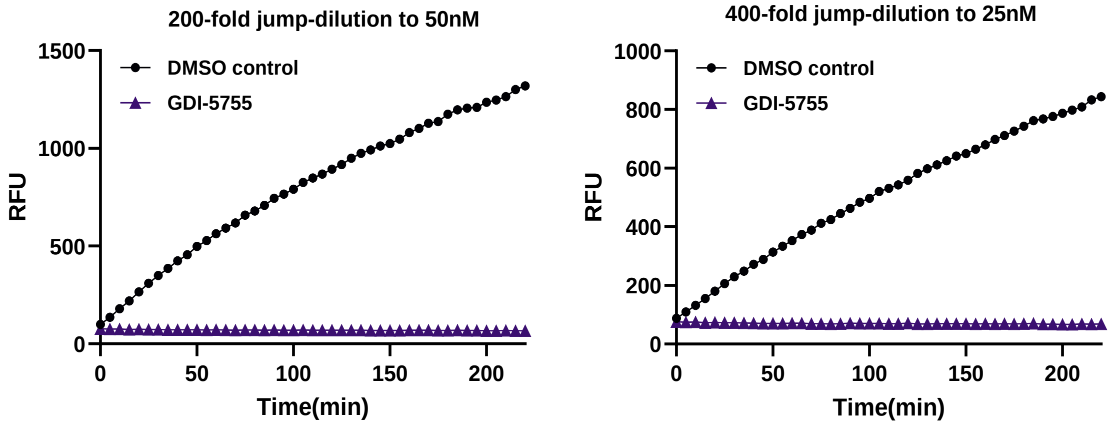

Figure S3. Progress curves of the peptidase reaction in the jump dilution experiment. A mixture of ClpP1P2 and GDI-5755 (or DMSO), each at 10 μM, was preincubated for ~1 hour. The mixture was diluted 200-fold or 400-fold with reaction buffer and substrate to initiate the reaction.

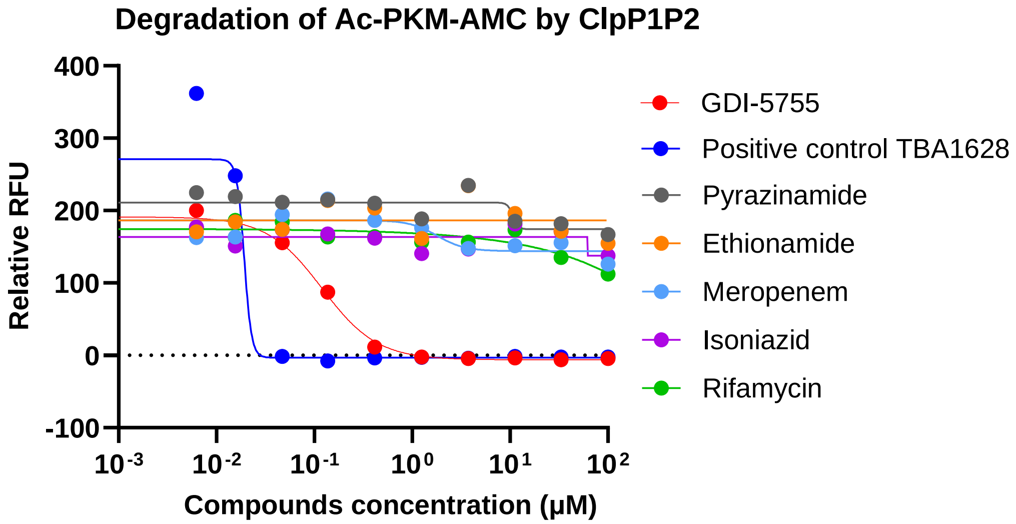

Figure S4. Testing of anti-TB drugs in the ClpP1P2 peptidase assay. Data points represent the averages of three independent replicates, and error bars indicate the standard errors of the mean (SEM) derived from the three experiments performed in duplicates. None of the drugs tested can inhibit 50% of the peptidase activity at 100 μM.

Scheme 1: Synthesis of GDI-5755, GDI-5756 and GDI-5757

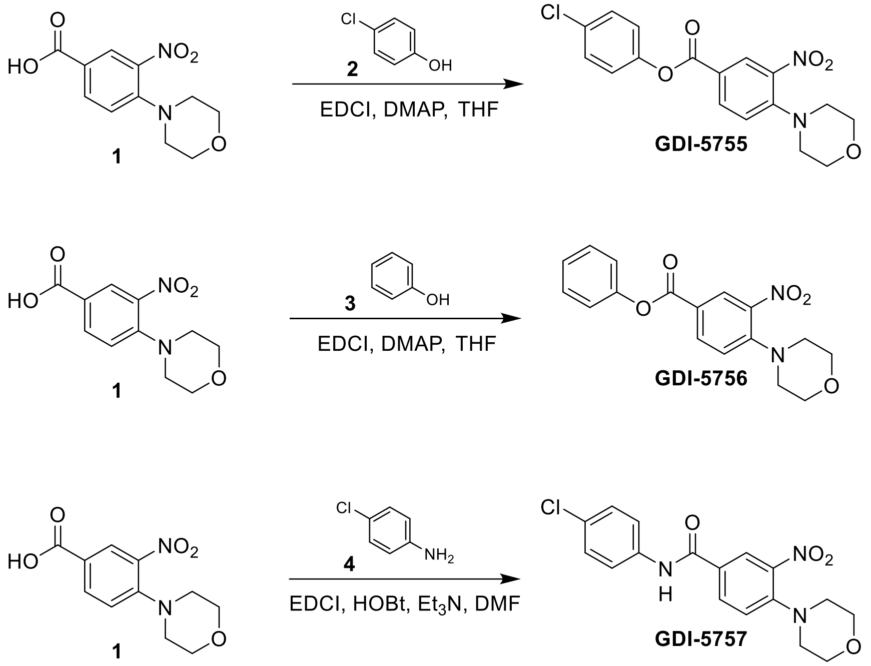

4-morpholino-3-nitrobenzoic acid was obtained with WO2009/43890, 2009 as reference; EDCI: n-(3-dimethylaminopropyl)-n'-ethylcarbodiimide hydrochloride; DMAP: 4-Dimethylaminopyridine; HOBt: 1-Hydroxybenzotriazole.

General procedure (A)

Synthesis of GDI-5755: Into a flask were added 4-morpholino-3-nitrobenzoic acid (**1,** 1.2 eq), 4-chlorophenol (**2**, 1 eq), EDCI (1.5 eq) and DMAP (2 eq), followed by addition of THF. The system was quenched with water after the mixture was stirred overnight at RT, extracted by ethyl acetate, and concentrated under reduced pressure. The residue was purified by column chromatography on silica gel eluting with petroleum ether/ ethyl acetate to give yellow solid 4-chlorophenyl 4-morpholino-3-nitrobenzoate. LCMS (purity: 99%, Ms: 362.9 [M+H]+).1H NMR (400MHz, DMSO) δ = 8.48 (d, J=2.4 Hz, 1H), 8.17 (dd, J=8.8, 2.4 Hz, 1H), 7.53 (dd, J=6.4, 2.0 Hz, 2H), 7.43 (d, J=8.8 Hz, 1H), 7.34 (dd, J=8.8, 2.0 Hz, 2H), 3.73 (t, J=4.8 Hz, 4H), 3.22 (t, J=4.8 Hz, 4H).

Synthesis of GDI-5756: Into a flask were added 4-morpholino-3-nitrobenzoic acid (**1,** 1.2 eq), phenol (**3**, 1 eq), EDCI (1.5 eq) and DMAP (2 eq), followed by addition of THF. The system was quenched with water after the mixture was stirred overnight at RT, extracted by ethyl acetate, concentrated under reduced pressure. And the residue was purified by column chromatography on silica gel eluting with petroleum ether/ ethyl acetate to give yellow solid phenyl 4-morpholino-3-nitrobenzoate. LCMS (purity: 98%, Ms: 328.9 [M+H]+).1H NMR (400MHz, DMSO) δ = 8.48 (d, J=2.0 Hz, 1H), 8.19 (dd, J=6.0, 2.0 Hz, 1H), 7.43 – 7.32 (m, 3H), 3.73 (t, J=4.4Hz, 4H), 3.22 (t, J=4.4 Hz, 4H).

Synthesis of GDI-5757: Into a flask were added 4-morpholino-3-nitrobenzoic acid (**1,** 1.2 eq), 4-chloroaniline (**4**, 1 eq), EDCI (1.5 eq), HOBt (1.5 eq) and Et_3N (3 eq), followed by addition of DMF. The system was quenched with water after the mixture was stirred overnight at RT, extracted by ethyl acetate, concentrated under reduced pressure. And the residue was purified by column chromatography on silica gel eluting with petroleum ether/ ethyl acetate to give yellow solid 4-chlorophenyl 4-morpholino-3-nitrobenzoate. LCMS (purity: 98%, Ms: 361.9 [M+H]+).1H NMR (400MHz, DMSO) δ = 10.39 (s, 1H), 8.46 (d, J=2.0 Hz, 1H), 8.15 (dd, J=6.0, 2.0 Hz, 1H), 7.79 (dd, J=6.8, 2.0 Hz, 1H), 7.45 – 7.37 (m, 3H), 3.72 (t, J=4.4Hz, 4H), 3.13 (t, J=4.4 Hz, 4H).

Additional methods

Peptide labeling and fractionation

The peptides were generated by digesting samples with trypsin enzyme, which was added at a ratio of 1:20 ratio (enzyme: substrate) and incubated overnight at 37 ℃. Extracted the digested peptide solution for desalination, followed by freeze-drying of the peptide solution. Then peptide samples were dissolved in 0.5 M TEAB and mixed with 8-plex iTRAQ labeling reagents. Subsequently, the mixture was incubated at room temperature for 2 hours. The labeling reaction was terminated by the addition of two volumes of water. The resultant labeled peptides from all 8 samples were mixed and dried under vacuum. The Shimadzu LC-20AB liquid phase system, equipped with a 5μm 4.6 x250mm Gemini C18 column, was utilized for liquid phase separation. Peptide samples, reconstituted with mobile phase A (5% ACN pH 9.8), were injected and eluted at a flow rate of 1mL/min using the following gradients: 5% mobile phase B (95% ACN, pH 9.8) for 10 minutes, followed by an increase from 5% to 35% mobile phase B over 40 minutes, a rapid increase from 35% to 95% mobile phase B in 1 minute, mobile phase B for 3 minutes, and finally returning to 5% mobile phase B for 10 minutes. The elution peak was monitored at 214nm, with one component collected per minute. Twenty fractions were obtained based on the chromatographic elution peak map and subsequently freeze-dried.

LC-MS analysis of labeled peptides

The dried peptide samples were dissolved in 15 μl of mobile phase A (2% ACN, 0.1% formic acid in water) and centrifuged at 20,000g for 10 min. The supernatant was then injected into a Thermo UltiMate 3000 UHPLC system. Separation occurred in a self-packed C18 column (75μm internal diameter, 3μm particle size, 25cm length) after initial enrichment and desalting in a trap column. The flow rate was set at 300nL/min, following this gradient: 0~5min, 5% mobile phase B (98% CAN,

0.1% formic acid); 5~45 minutes, mobile phase B increased linearly from 5% to 25%; 45~50 min, mobile phase B increased from 25% to 35%; 50~52 minutes, mobile phase B increased from 35% to 80%; 52~54 min, 80% mobile phase B; 54~60 minutes, 5% mobile phase B. The separated components were directly connected to the mass spectrometer at the end of the nanoliter liquid phase separation. The liquid chromatography-separated peptides were ionized using a nanoESI source and analyzed using a Q-Exactive HF X tandem mass spectrometer (Thermo Fisher Scientific, San Jose, CA) in Data Dependent Acquisition (DDA) mode. Key parameters included an ion source voltage of 1.9kV, MS1 scanning range from 350 to 1500 m/z, and a resolution of 60,000. MS2 scans began at an m/z of 100 with a resolution of 15,000. Fragmentation occurred using High Collision Dissociation (HCD), and fragment ions were detected in the Orbitrap. For MS2 fragmentation, ions with a charge of 2+ to 6+ and the top 30 parent ions with a peak intensity exceeding 10,000 were selected. Dynamic exclusion was set to 30 seconds, and AGC was configured as follows: MS1 3E6, MS2 1E5.

Peptide identification and quantitation

MS/MS spectra were searched against the *Mycobacterium bovis* (strain BCG/Pasteur 1173P2) reference proteome, downloaded from UniProt (https://www.uniprot.org/proteomes/). Additionally, protein databases like NCBInr and SwissProt were also used for protein identification. The IQuant software was used for quantitative analysis of labeled peptides with isobaric tags. This software integrates Mascot Percolator, a machine learning method, to improve the reliability of database search results. Peptide-spectrum matches (PSMs) were pre-filtered at a 1% false discovery rate (FDR) at the PSM level. Identified peptide sequences were then assembled into a set of confident proteins using the parsimony principle. To control false positives at the protein level, a protein FDR of 1% was estimated using the Picked protein FDR strategy. Thus, parameters including score, protein coverage, and the number of unique peptides were obtained for each protein. Additionally, proteins exhibiting a fold change exceeding 25% (with a ratio of either <0.75 or > 1. 25) were considered significantly expressed on the protein level. A total of 2932 proteins of BCG can be detected by this method: 1937 in the day 2 sample and 1875 in the day 3 sample have a $P < 0.05$.
